# Building a stronger future together: a message from the new Editor-in-Chief

**DOI:** 10.1093/rap/rkaf021

**Published:** 2025-03-17

**Authors:** Mwidimi Ndosi

**Affiliations:** School of Health and Social Wellbeing, College of Health, Science and Society, University of the West of England, Bristol, UK; Rheumatology Department, University Hospitals Bristol and Weston NHS Foundation Trust, Bristol, UK

I am honoured to assume the role of Editor-in-Chief of *Rheumatology Advances in Practice (RAP).* This appointment is both a personal honour and an opportunity to contribute to a journal that shapes rheumatology research and patient care.

As I assume this position, I am mindful of the remarkable legacy left by my predecessors. Professor Richard Watts, the founding Editor-in-Chief, envisioned a platform that would bridge the gap between research and clinical practice. Dr Ai Lyn Tan, my immediate predecessor, built upon this vision with exceptional leadership, strengthening *RAP*’s reputation as an essential resource for rheumatology and musculoskeletal care professionals. I am inspired by their achievements and committed to upholding and advancing the mission of our journal.

*RAP* is more than just a repository of research—it is a dynamic and inclusive community of healthcare professionals dedicated to improving patient lives. This community brings together rheumatologists, nurse specialists, physiotherapists, occupational therapists, podiatrists, pharmacists and psychologists, each playing a vital role in advancing patient care. Through the publication of high-quality research, the journal equips practitioners with the knowledge to make informed decisions and deliver the highest standard of care.

My vision for the journal is both ambitious and collaborative, building on our strong foundation while embracing new opportunities for growth and innovation. One of the most valuable aspects of *RAP* is its collaborative spirit. Our dedicated reviewers, editorial board and authors are driving the success of our journal through commitment to high-quality research.

As we move forward, I am committed to expanding our global reach and ensuring diverse perspectives are represented. Patients and practitioners face unique challenges, and our journal has a responsibility to reflect these realities. This will enrich our content, foster innovation and drive meaningful progress. To support this, we will prioritise mentoring initiatives that equip early-career researchers with essential peer review skills and a deeper understanding of academic publishing. These initiatives will also extend to health professionals in rheumatology, who play a crucial role in translating research into clinical practice.

The rapid pace of innovation in rheumatology demands that *RAP* remain at the forefront of emerging trends and technologies. From novel biologics and small molecules to the transformative potential of digital health and artificial intelligence, our journal must continue to be a platform for cutting-edge research and critical discussions that shape the future of rheumatology. However, innovation is meaningful only when it is accessible and widely understood. As an Open Access journal, we are committed to making *RAP* more inclusive for a broader audience. We encourage lay summaries in our articles to ensure that complex research is presented in a clear and concise manner, making it understandable to patients, caregivers and the general public. Additionally, our expanded social media presence will encourage direct dialogue between researchers, practitioners and the wider community. These efforts ensure *RAP* serves both professionals and those living with rheumatic and musculoskeletal conditions. Ultimately, our goal is to ensure that research evidence reaches, educates and inspires as many people as possible while driving progress in the field.

This new chapter for *RAP* is not mine alone—it belongs to all of us. I am deeply grateful for the trust and support of my predecessors, colleagues, the British Society for Rheumatology, Oxford University Press and the wider rheumatology community, who entrust us with their research. The passion and dedication to this field are truly inspiring. Together, we will build on the remarkable legacy of this journal and drive innovation in rheumatology. Thank you for your ongoing commitment to *RAP* and, most importantly, to improving the lives of patients.

## Data Availability

No new data were generated or analysed in support of this article.

